# Combined Hamartoma of the Retina and Retinal Pigment Epithelium in a Patient with Gorlin Syndrome: Spontaneous Partial Resolution of Traction Caused by Epiretinal Membrane

**DOI:** 10.1155/2016/2312196

**Published:** 2016-08-09

**Authors:** José L. Sánchez-Vicente, Miguel Contreras-Díaz, Trinidad Rueda, Enrique Rodríguez de la Rúa-Franch, Fredy E. Molina-Socola, Cristina Vital-Berral, Asunción Alfaro-Juárez, Fernando López-Herrero, Ana Muñoz-Morales

**Affiliations:** Department of Ophthalmology, Virgen del Rocio University Hospital, 41013 Seville, Spain

## Abstract

*Purpose*. To describe the case of spontaneous resolution of epiretinal membrane in a patient with Combined Hamartoma of the Retina and Retinal Pigment Epithelium (CHR-RPE), in the clinical context of Gorlin Syndrome (GS).* Methods*. Observational case report of a 12-year-old female patient is presented. The diagnosis of CHRRPE was made by OCT and fundus examination, which showed a mound of disorganized tissue originating from retina and retinal pigment epithelium. Epiretinal membrane (EM) was also detected. Genetic study was performed to confirm the diagnosis of GS.* Results*. The patient was observed for 39 months, showing spontaneous resolution of the traction caused by the EM and improvement in visual acuity (VA), which was 20/80 at initial presentation, rising to 20/40 after follow-up period.* Conclusions*. The presence of EM in CHR-REP is a cause of reduction of visual acuity. Management of this condition is controversial; however, we would like to highlight that spontaneous resolution of the traction caused by EM is possible, resulting in recovery of VA.

## 1. Introduction

Gorlin Syndrome (GS), also known as Nevoid Basal Cell Carcinoma Syndrome, was characterized for the first time in 1960 by Gorlin and Goltz, who described the association of basal cell carcinoma, odontogenic keratocysts, and bifid ribs [1].

The disease is a hereditary condition caused by mutation in PTCH1 gene and is transmitted as an autosomal dominant trait with complete penetrance and variable expressivity. PTCH1 gene is located in the long arm of chromosome 9 (9q22.3) and encodes a membrane receptor which has a fundamental function in controlling growth and development of normal tissues. This gene acts as a tumor suppressor [2].

GS is rare; it has an estimated prevalence from 1/57,000 to 1/256,000. There is no sexual predilection. Ocular manifestations appear in 15–22% of patients including hypertelorism, strabismus, myelinated fibres, and epiretinal membranes (ERM) [3].

We present the case of a 12-year-old female patient with GS and Combined Hamartoma of the Retina and Retinal Pigment Epithelium (CHR-RPE), with 39 months of follow-up, in which spontaneous resolution of the traction caused by the ERM was observed.

## 2. Case Report

A 12-year-old female patient was referred to our retina service due to decrease in visual acuity (VA) in her right eye (RE).

**Figure 1:**
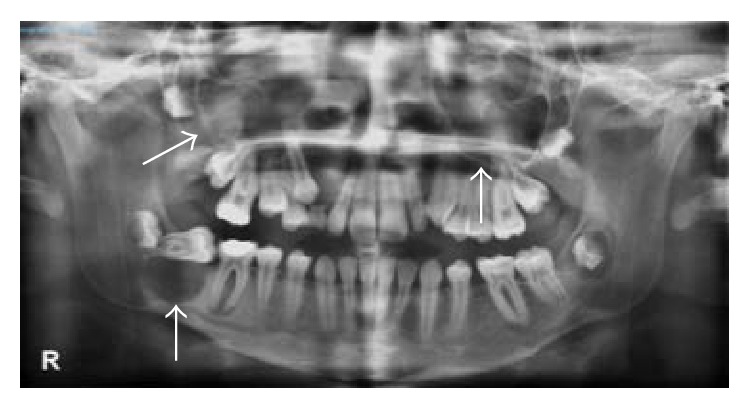
Orthopantomography. Odontogenic cysts (arrows).

She had an ophthalmic history of strabismus, hyperopia, and amblyopia in her RE. Her mother had been operated on because of a skin cancer and maxillary cysts. The patient presented multiple odontogenic cysts (Figure 1) and scoliosis, and she had been previously studied in Neuropediatrics because of macrocephaly. Her intellectual capacity was normal.

Clinical examination showed VA in right eye 20/80 and left eye 20/20. Anterior segment evaluation showed no alteration, and intraocular pressure was normal in both eyes.

**Figure 2:**
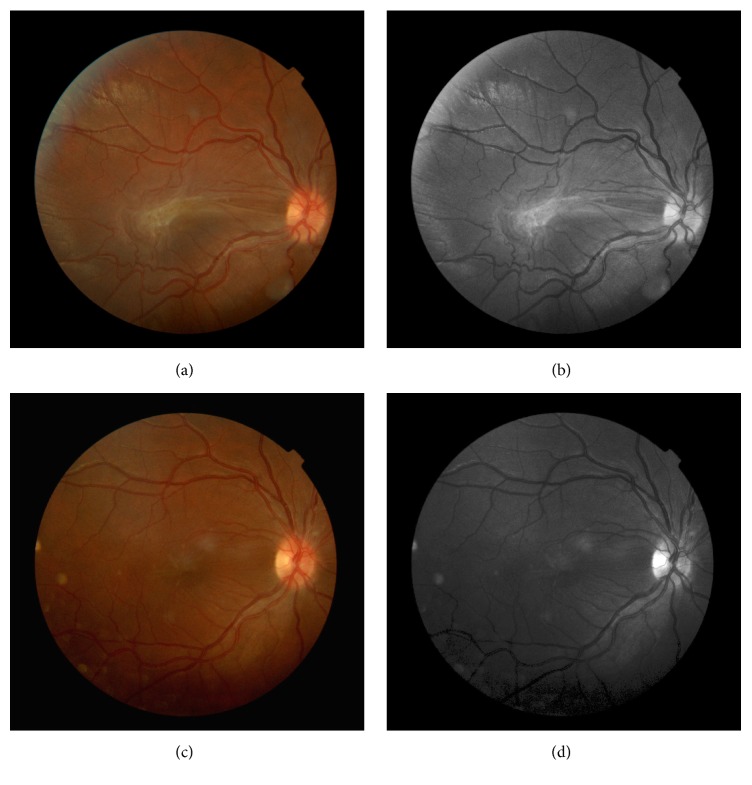
(a) Color retinography of the right eye and (b) reed free image at initial presentation. An elevated macular lesion, slightly pigmented, with vascular tortuosity and associated epiretinal membrane, is observed. (c) Retinography of the right eye and (d) reed free image 18 months after follow-up, showing resolution of the traction caused by the epiretinal membrane, reduction of vascular tortuosity, and retinal folds.

Fundus examination revealed an elevated lesion in the temporal region of the macula, white-greyish coloured, with associated vascular distortion, which caused important traction in macular area (Figure 2).

**Figure 3:**
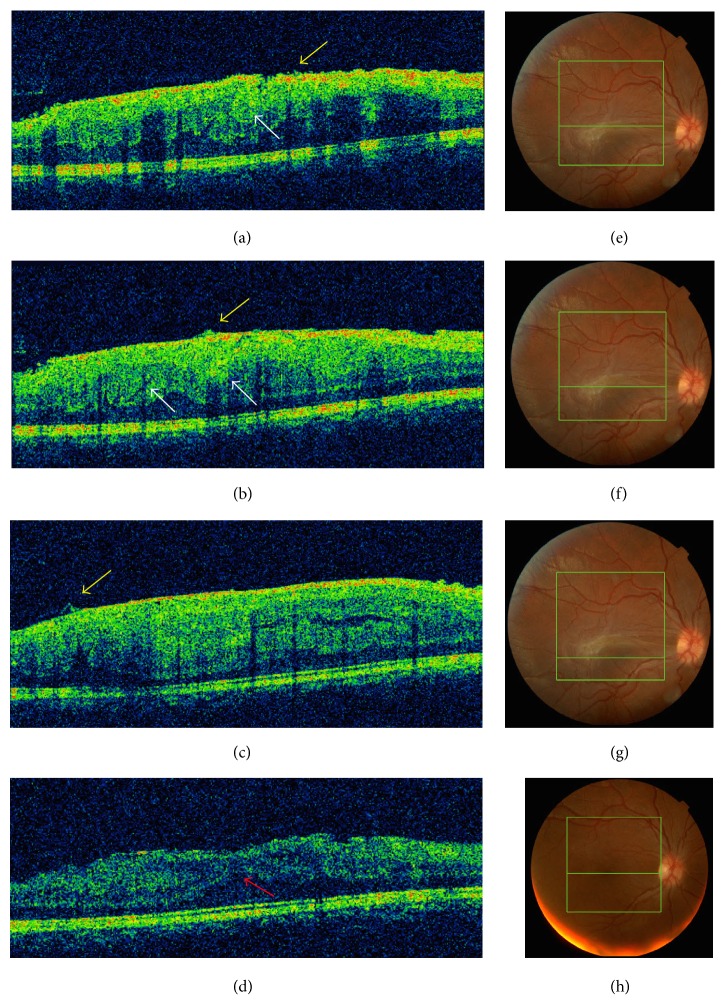
(a, b, and c) OCT at initial presentation and (d) 18 months after follow-up. (e, f, g, and h) Fundus photography corresponding to each OCT. (a, b, and c): The lession reveals disorganization of normal retinal layers, with hyperrfletive surface and hyporreflective shadowing. White arrows: full thickness retinal folds with superficial and deep retinal distortion. Yellow arrows: peaks in the inner retina correspondig to the traction caused by the epiretinal membrane. (d): OCT 18 months after follow-up, showing relief of the macular traction and reduction of macular thickness. Retinal layers can be identified. Red arrow: outer nuclear layer.

Optical Coherence Tomography (OCT) scan showed disorganization of the normal retinal layers, a hyperreflective mass with hyporeflective shadowing, and epiretinal membrane, leading to high distortion of the macular region (Figure 3).

**Figure 4:**
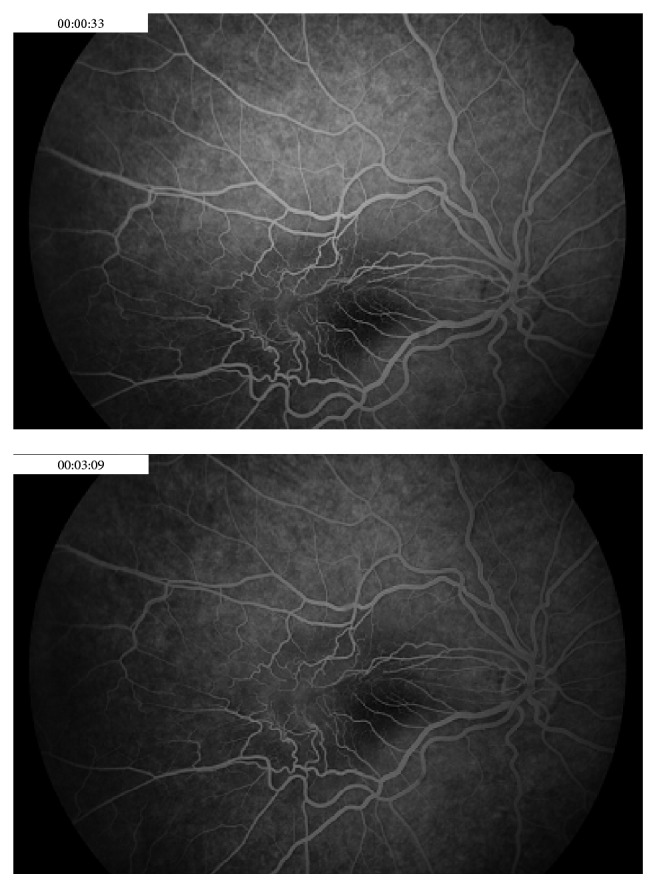
Fluorescein angiography of the right eye, prior to spontaneous resolution. Severe vascular tortuosity with mild leakage after three minutes of fluorescein injection.

Fluorescein angiography showed retinal vascular tortuosity with early hyperfluorescence and mild leakage after three minutes of fluorescein injection (Figure 4).

With all these tests, diagnosis of CHR-RPE was made, and GS was suspected because of the family and personal history. Genetic study of the patient and her mother was obtained, and mutation c.3152G>a was detected in heterozygosis in PTCH1 gene.

Surgical management of the ERM associated with the retinal mass was offered, but the parents refused, because of a stable VA since the beginning of the study. Therefore we decided observation of the patient.

18 months after follow-up, in OCT examination, spontaneous partial resolution of the traction caused by the ERM was observed, together with a recovery in VA in right eye, which now was 20/40 (Figures 2 and 3).

During the follow-up, the patient underwent bilateral curettage of the maxillary sinus because of chronic sinusitis associated with odontogenic cyst. In addition, a millimetric basal cell carcinoma was detected and observed every 3 months.

Multidisciplinary approach has been necessary to assess this case, including paediatrics, maxillofacial surgery, dermatology, and ophthalmology.

## 3. Discussion

GS is a rare multisystemic disease associated with skin, skeletal, craniofacial, neurological, oropharyngeal, genitourinary, and cardiac abnormalities [2]. Diagnostic criteria were first defined by Evans et al. in 1993 [4], modified by Kimonis et al. in 1997 [5] and later by Bree et al. in 2011 [6] (see below).

*Diagnostic Criteria of Gorlin Syndrome*. A suspected diagnosis of BCNS^*∗*^ could be reasonably considered based on the findings of (1) one major criterion and molecular confirmation, (2) two major criteria, or (3) one major and two minor criteria.

*Major Criteria*. Major criteria were as follows:

 BCC^†^ prior to 20 years of age or excessive numbers of BCC out of proportion to prior sun exposure and skin type.
 Odontogenic keratocysts of the jaw prior to 20 years of age.
 Palmar or plantar pitting.
 Lamellar calcification of the falx cerebri.
 Medulloblastoma, typically desmoplastic.
 First-degree relative with BSCS.

*Minor Criteria*. Minor criteria were as follows:

 Rib anomalies.
 Other specific skeletal malformations and radiologic changes (i.e., vertebral anomalies, kyphoscoliosis, short fourth metacarpals, and postaxial polydactyly).
 Macrocephaly.
 Cleft/lip palate.
 Ovarian/cardiac fibroma.
 Lymphomesenteric cysts.
 Ocular abnormalities (i.e., strabismus, hypertelorism, congenital cataracts, glaucoma, and coloboma).

*∗* is basal cell nevoid syndrome and † is basal cell carcinoma, taken from [6].

Among the ocular manifestations of GS, CHR-RPE is one of the possible presentations. There is only one report in the literature about this association [7]. CHR-RPE is a presumed congenital benign intraocular tumor characterized by a mound of disorganized glial, vascular, and melanocytic tissue originating from the retina and retinal pigment epithelium. They are commonly associated with ERMs producing retinal distortion, macular dragging, and ultimate vision loss [8, 9].

Some authors have concluded that the ERM is a differentiated entity going in conjunction with the CHR-RPE, but extrinsic to the retina [9], and some reports have been published about the positive effect of surgery in patients with ERM and CHR-RPE. However, there are no established criteria to determine how intrinsic the ERM is to the retina, and to date, the surgery of CHR-RPE remains controversial in terms of recovery of VA [10, 11].

In our patient, we decided to postpone the surgery, because VA was stable since the first clinical examination, assuming that the decrease in VA was due to the CHR-RPE. However, during the follow-up, spontaneous relief of the traction caused by the ERM was observed, with improvement in VA. That suggests that early surgery in selected cases could be of benefit in terms of recovery of VA, which have been concluded in some other works [9, 11, 12].

GS is a rare multisystemic disease probably infradiagnosed. Genetic study is of special interest in the diagnosis of this condition when suspected. Patients require a multidisciplinar approach, given the wide spectrum of affected systems, some of them of important gravity.
